# Optimisation of the co-combustion of meat–bone meal and sewage sludge in terms of the quality produced ashes used as substitute of phosphorites

**DOI:** 10.1007/s11356-020-11022-5

**Published:** 2020-10-14

**Authors:** Zygmunt Kowalski, Marcin Banach, Agnieszka Makara

**Affiliations:** 1 Mineral and Energy Economy Research Institute Polish Academy of Sciences, Wybickiego 7, 31-261 Krakow, Poland; 2 Faculty of Chemical Engineering and Technology, Cracow University of Technology, Warszawska 24, 31-155 Krakow, Poland

**Keywords:** Meat–bone meal, Sewage sludge, Thermal utilisation, Ash quality, Phosphorite

## Abstract

To obtain a high-quality phosphorus raw material comparable in quality to the best phosphorites used in the fertiliser industry, an analysis was carried out to determine the optimal mass proportions of a meat–bone meal and sewage sludge mixture to be used in a co-combustion process. The ashes obtained contained hydroxyapatite that could be considered a high-quality substitute for phosphorites, with an average P_2_O_5_ content of 33.5%. These ashes do not contain fluorine compounds, cadmium content is at trace levels and they do not contain the radioactive compounds that are present in trace amounts in all phosphates. The developed process is an example of a production complex using cleaner technologies and circular economy principles on a microeconomic scale.

## Introduction

In the European Union, 18 Mt/year of meat waste is generated and is mainly processed into meat–bone meal (MBM). The EU produces 4.5 Mt/year of MBM, used typically as biofuel (Hiromi Ariyaratne et al. 2015; Gulyurtlu et al. 2005) and mineral–organic fertilisers (Möller 2015; Lucid et al. 2013). Studies (Nogalska and Zalewska 2013; Černý et al. 2012) indicate that MBM is a valuable source of phosphorus for grain crops and, in particular, silage maize and increased maize productivity.

Thermal treatment of waste from the meat industry in a large-scale rotary kiln was presented by Staroń et al. (2017). The main results of research into combustion, pyrolysis and gasification of MBM (Cascarosa et al. 2012) showed that the products could be used as fertiliser (solid product) and as fuel (gas and liquid products). The work aimed at reviewing the most significant studies into the potential application of the products obtained in these thermochemical processes that contain hydroxyapatite. Thermal decomposition of MBM was investigated by Conesa et al. (2003). Characteristics of industrial and laboratory MBM ashes and their potential applications were presented by Coutand et al. (2008). Hu et al. (2017) dealt with hydroxyapatite and its ferroelectric properties that may be interesting for biological/biomedical applications.

Under the European project COPOWER, three co-combustion scenarios (Sc) were studied: Sc0 − combustion of coal, Sc1 − combustion of coal + sewage sludge (SS) + MBM and Sc2 − coal + SS + wood pellet (WP). From the environmental point of view, Sc0 was the worst scenario and Sc1 the best, due mainly to the reduction in GHG emission, eutrophication chemical species and ozone-depleting gases. From the socio-economic point of view, Sc0 was the worst scenario Sc1 the best, due to the lowest cost of electricity production and negative cost of avoided emissions (Morais et al. 2011).

In Hiromi Ariyaratne et al. (2015), MBM and coal combustion in a rotary cement kiln were simulated and the results compared. The equilibrium temperature of the MBM combustion products was 300 K lower than those of coal combustion, due mainly to the higher air demand, higher ash and moisture content and poor char burnout (83%) of MBM. Fluidised bed co-combustion of MBM with coal were also investigated by Fryda et al. (2006) and Gulyurtlu et al. (2005).

The granulation of meat and bone meal, straw and SS and incineration of granulated product were investigated by Kowalski et al. (2012). The incineration of MBM/feather mixtures was tested by Staroń et al. (2015). The thermal conversion of granules from feathers, MBM and poultry litter to ash with fertilising properties were proposed by Staroń et al. (2017a). The work by Heikkinen et al. (2008) described computational fluid dynamics CFD simulation and experimental validation of co-combustion of chicken litter and MBM with pulverised coal in a flow reactor. According to Wzorek (2015), fuels based on the combined use of MBM and SS fulfil the requirements and may be applied as a substitute for coal in clinker cement burning.

The evaluation of the four alternative SS management strategies (Bertanza et al. 2016) led to the following preference order: agricultural use ≫ incineration > cement kiln ≅ wet oxidation. The assessments of the leading SS disposal (volume reduction) and energy recovery routes such as incineration, pyrolysis and gasification were made by Raheem et al. (2018). The thermal treatment of SS and the characteristics of ashes from its incineration were presented briefly by Tarko (2016).

These solutions are an example of the application in industrial practice of circular economy (CE) systems, emphasising the possibility of using to the greatest possible extent the value contained in materials (Asif et al. 2016). According to the CE, combustion for energy recovery is also a useful option. In this way, the product life cycle value chain retains the highest possible value and quality as long as possible and is also as energy efficient as it can be (Ghisellini et al. 2016; Bilitewsky 2012).

For general use in the fertiliser industry, phosphate rock or its concentrates preferably have levels of 30% P_2_O_5_, reasonable amounts of CaCO_3_ (5%) and < 4% combined iron and aluminium oxides (Filippelli 2011; Blatt and Tracy 1996). Worldwide, the resources of high-grade ore are declining, and the beneficiation of lower-grade ores occurs through washing, screening, de-liming, magnetic separation or flotation (Hogan 2011; Zapata and Roy 2004).

The basic criterion regarding the quality of phosphate rock used in the production of phosphoric acid is content (Eq. 1) P_2_O_5_ or BPL (bone phosphate of lime; Becker 1989):

1 $$ \mathrm{BPL}=2.1852\cdot {\mathrm{P}}_2{\mathrm{O}}_5 $$

Phosphate rock with a content of 33–39% P_2_O_5_ (72–85 BPL) is considered to be of high quality. Medium-quality rock contains 30–33% P_2_O_5_ (65–72 BPL) and low-quality 26–30% P_2_O_5_ (57–65 BPL) (International Fertilizer Development Center 1998; Podolak 2017).

For processing of phosphorites into phosphoric(V) acid and NP or NPK fertilisers, the sum of aluminium, iron and magnesium oxides (Becker 1989) is of particular importance. The high value of this sum can lead to poor chemical properties of superphosphates, problems during the fertiliser production process and drying of products made from phosphoric acid obtained from such phosphates. For processing of phosphorites into phosphoric(V) acid and NP or NPK fertilisers, the sum of aluminium, iron and magnesium impurities determined by the Minor Element Ratio (MER) index (Becker 1989; BAT 2000) is used (Eq. 2):

2 $$ \mathrm{MER}=100\times \left(\sum \left({\mathrm{Al}}_2{\mathrm{O}}_3,{\mathrm{Fe}}_2{\mathrm{O}}_3,\mathrm{MgO}\right)\right)/{\mathrm{P}}_2{\mathrm{O}}_5\left(\%\right) $$

If the ∑(Al_2_O_3_, Fe_2_O_3_, MgO) content increases > 3% by mass, there are problems associated with precipitation during concentration, cooling and storage of phosphoric acid. High concentrations of iron and aluminium during storage may cause acid compounds containing digestible forms of P_2_O_5_ to convert into compounds with the non-digestible form of P_2_O_5_. Typically, the acceptable level of impurities in H_3_PO_4_ used to produce fertilisers is given by Eq. 3 (BAT 2000; Podolak 2017):

3 $$ 0.02\le \left({\mathrm{Fe}}_2{\mathrm{O}}_3+{\mathrm{Al}}_2{\mathrm{O}}_3\right)/{\mathrm{P}}_2{\mathrm{O}}_5\le 0.08 $$

Table 1 presents composition of phosphorite ores used for production of phosphoric acid and phosphorus fertilisers, including types of impurities important in these processes, calculated from Podolak (2017).

**Table 1:** Content of P_2_O_5_ and impurities in phosphorites (used as expected parameters for ashes from co-combustion of MBM and SS)

Phosphorite type	Content (%) or ratio (%)							
	P_2_O_5_	Al_2_O_3_	Fe_2_O_3_	(Fe_2_O_3_ + Al_2_O_3_)/P_2_O_5_	MgO	MER^a^	Cd (ppm)	*X*_Cd_ (mg Cd/kg P_2_O_5_)
Morocco–Togo	33.8	0.888	0.973	0.06	0.23	6.2	37	109
Tunisia–Jordan	29.8	0.423	0.287	0.02	0.33	3.5	13	44
Morocco–Syria	29.7	0.348	0.171	0.02	0.39	3.1	11	37
Tunisia–Syria	28.9	0.357	0.189	0.02	0.40	3.3	13	45
Tunisia–Syria	28.9	0.427	0.197	0.02	0.44	3.7	17	59
Syria	30.0	0.23	0.17	0.01	0.45	2.8	7	23
Tunisia	29.1	0.47	0.36	0.03	0.65	5.1	33	113
Algiers	29.9	0.32	0.44	0.03	0.95	5.7	16	54
Togo	33.5	1.08	1.33	0.07	0.17	7.7	49	146
Morocco	31.6	0.30	0.20	0.02	0.45	3.0	16	51
Egypt	28.9	0.49	1.70	0.08	0.30	8.6	9.5	33
Senegal Matam	32.3	1.19	0.80	0.06	0.38	7.3	11	34
Senegal Lam-Lam	33.3	3.59	1.62	0.16	0.38	16.8	52	156

^a^Minor element ratio, MER = 100 × (∑(Al_2_O_3_, Fe_2_O_3_, MgO))/P_2_O_5_ (%)

The BPL value of phosphorites could be considered rather as medium-quality raw materials, with P_2_O_5_ in the range 28.9–33.8%, a (Fe_2_O_3_ + Al_2_O_3_)/P_2_O_5_ value in range 0.02–0.16 and MER index in range 3.0–16.8%. All phosphorites also contain typically (%): 3.5–4.0 F, 48.0–51.0 CaO, 2.4–5.0 SiO_2_ and 0.32–2.9 SO_3_ (Becker 1989).

Co-combustion of MBM and SS for energy recovery from these wastes and the use of the obtained ash containing hydroxyapatite as alternative raw material for the production of phosphorus compounds have been proposed (Stokłosa et al. 2019; Kowalski and Makara 2017). The goal of the research was to determine the optimal mass proportions of co-combusted MBM and SS that would allow obtaining ash with a quality comparable or better than that in phosphorite ores used in Poland for the production of phosphoric acid.

## Materials and methods

### Characteristic of ash after combustion of MBM/SS mixture

**Fig. 1:**
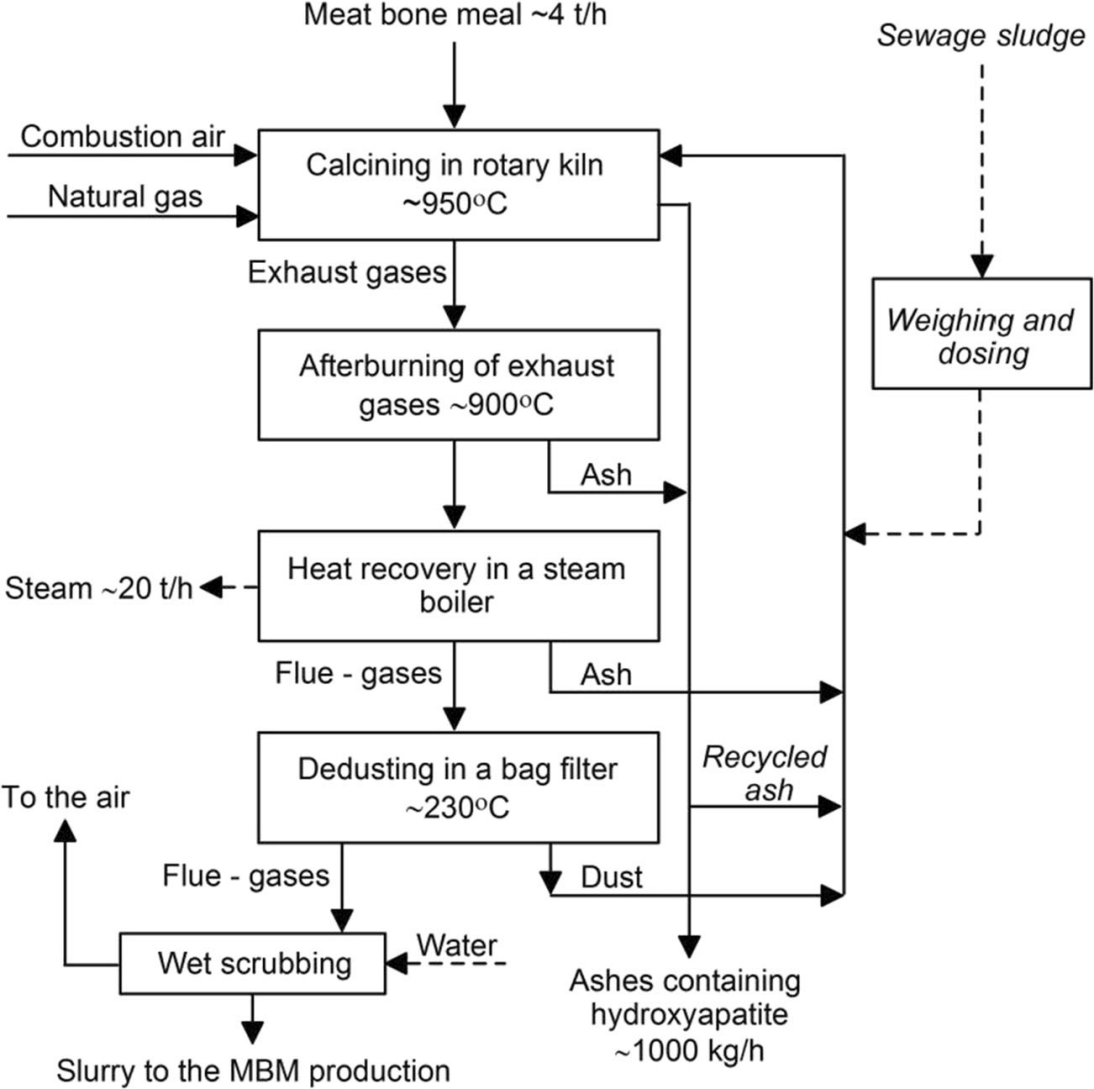
Flow-sheet of the MBM/SS co-combustion unit

MBM and SS were come from Farmutil Inc. the biggest Polish MBM producer, having own large MBM wastewater treatment unit. MBM combustion unit (capacity 4 t/h) started in 2019. The co-combustion of MBM and sewage sludge dry mass (SSDM) was carried out on experimental facility. The schematic diagram of the MBM thermal processing is shown in Fig. 1 (Kowalski and Makara 2017; Stokłosa et al. 2017).

Calcining of MBM in a rotary kiln includes complete incineration of organic part of the MBM in a two-stage process carried out with at least 20% excess air compared to the stoichiometric amount necessary for the complete oxidation of the organic materials. In a co-current rotary kiln, thermal operations and reactions take place, including drying-degassing, incineration and combustion of carbonised organic matter and calcining of calcium phosphates. MBM is dosed with screw conveyors into a co-current rotary kiln. The heat of combustion of MBM is estimated to be 18.5 MJ/kg.

The ash is collected from the rotary kiln and after-burning chambers and transported by screw conveyors to the storage. The possibility is foreseen of recycling the ash into the rotary kiln and the weighing and dosing of the SS. The exhaust gas from the rotary kiln is additionally burned in the after-burning chamber. The heat of the exhaust gases is used to produce process steam in the boiler. Flue gas after the steam boiler is de-dusted in a bag filter, next cleaned using wet scrubbing and then directed to the air.

MBM and SS require calcining at temperatures up to 950 °C. From the thermal decomposition of 1000 kg of MBM, 250 kg of ash was obtained, containing almost pure hydroxyapatite Ca_5_(PO_4_)_3_OH. The hydroxyapatite crystals obtained were well formed and not fused under these conditions. X-ray analysis also showed the presence of small amounts of Ca_3_(PO_4_)_2_, CaCO_3_, SiO_2_ and Fe_2_O_3_ in the ash. The product contained 16–18% P (36.6–41.2% P_2_O_5_) and on average 32.5% Ca. The phosphorus content in the product is therefore higher than in the compared phosphorites (see Table 1), where it is up to 33.8% P_2_O_5_.

The heat of combustion of SSDM is 16.5 MJ/kg, and ignition losses at 550 °C are 64%. X-ray analysis showed that the basic crystalline phases of the ash from calcining of sewage sludge at 950 °C are quartz, anhydrite, iron calcium phosphate and haematite. There are also small amounts of CaCO_3_ and iron phosphate (Lynn et al. 2015; Tarko 2016).

Table 2 presents the contents of P_2_O_5_ and important impurities in the production of phosphoric acid and phosphorus fertilisers in meat–bone meal ash (MBMA) and sewage sludge ash (SSA).

**Table 2:** Content of P_2_O_5_ and basic impurities in MBM, MBMA, SSDM and SSA

Phosphorite type	Content (%) or ratio (%)							
	P_2_O_5_	Al_2_O_3_	Fe_2_O_3_	(Fe_2_O_3_ + Al_2_O_3_)/P_2_O_5_	MgO	MER^a^	Cd (ppm)	*X*_Cd_ (mg Cd/kg P_2_O_5_)
MBM	9.6	0.0	0.005	0.0005	0.13	1.405	0.002	0.02
MBMA	41.0	0.0	0.014	0.0003	0.33	0.839	0.014	0.03
SSDM	3.55	2.02	5.471	2.110	0.39	222.03	2.9	81.7
SSA	12.8	7.28	19.70	2.110	1.40	222.03	10.44	81.7

^a^Minor element ratio, MER = 100 × (∑(Al_2_O_3_, Fe_2_O_3_, MgO))/P_2_O_5_ (%)

MBM contains typically the following (%): 8.0–10.0 CaO, 6.0–8.0 N, 0.5–0.6 SiO_2_, 0.01–0.02 F, 0.5–1.0 K and 1.5–2.0 Na. The content of heavy metals (As, Cu, Pb, Cr, Ni, Ag) is in range 0.001–0.0001% (Wilkosz-Język 2007). The results of the analysis indicate that MBMA has very high P_2_O_5_ content and is simultaneously a pure product containing very low amounts of iron and aluminium oxides (MER < 1), heavy metals and fluorine. Thus, it is easier to produce phosphoric acid and phosphoric fertilisers from MBMA, in particular without the use of very troublesome defluorination operations in industrial practice.

SSDM contains typically of the following (%): 3.5–4.0 CaO, 3.0–4.0 N, 8.0–10.0 SiO_2_ and 0.15–0.3 K + Na. The content of heavy metals (As, Cu, Pb, Cr, Ni, Ag) is in range 0.01–0.001%. SSA has low P_2_O_5_ content and very high iron and aluminium oxide contents (MER > 200), and therefore, it is not used directly for the production of phosphoric acid. However, heavy metal content in SSDM is very low. From 1000 kg of SS dry mass, 280 kg of ash was obtained (Rzepecki 2003; Piasta and Lukawska 2016).

### Statistical analysis

To obtain a high-quality phosphorus raw material comparable in quality to the best phosphorites used in the fertiliser industry, the co-combustion of MBM and SS would probably require a low addition of SS to MBM. For this reason, an analysis was carried out to determine the optimal mass proportions of MBM and SS for use in the co-combustion process.

Variables (Fe_2_O_3_ + Al_2_O_3_)/P_2_O_5_ and MER were calculated using variables that determine the content of oxide forms in the tested materials, as a combination of them. Dependent variables were P_2_O_5_, Fe_2_O_3_, Al_2_O_3_ and MgO contents. Mass fractions of MBM and SS were independent variables.

The response surface regression method was used for mixtures. Response surface regression models for mixtures are similar to second-degree factor regression systems; however, they lack free expression. The regression equation for the regression model of the response surface for mixtures, with two continuous explanatory variables MBM and SSDM, has the form of Eq. 4:

4 $$ Y={b}_1\mathrm{MBM}+{b}_2\mathrm{SSDM} $$

Model coefficients were determined for each dependent variable. In order to select the most favourable variables of the dependent values, the utility function values of the independent variables were used, where a value of 1 means the most favourable and 0 unfavourable. All experimental planning was performed using STATISTICA (version 10.0) (Hill and Lewicki 2007; StatSoft 2006).

From the point of view of Cd content, hydroxyapatite ash is a very advantageous raw material for phosphorus fertiliser production. The European Commission has proposed a regulation of increasingly stringent limits of Cd in fertilisers. An initial limit of 60 mg Cd/kg P_2_O_5_ will apply as soon as the regulation comes into force. A more stringent limit of 40 mg Cd/kg P_2_O_5_ will be phased in 3 years later, and the lowest limit of 20 mg Cd/kg P_2_O_5_ will come into force 9 years after the initial effective date (Regulation (EU) 2019). The Cd content in hydroxyapatite ash *X*_Cd_ = 0.17 mg Cd/kg P_2_O_5_ is much below of value for phosphate rock (*X*_Cd_ ranged from 23 to 156 mg Cd/kg P_2_O_5_; see Table 3), so the two parameters of Cd (ppm) and *X*_Cd_ cannot be dependent variables.

**Table 3:** Characteristic parameters of MBM and SS co-combustion products

No	Participation		Characteristic parameters of ashes from combustion of MBM and SS mixtures							
	MBM	SSDM	P_2_O_5_ (%)	Al_2_O_3_ (%)	Fe_2_O_3_ (%)	(Fe_2_O_3_ + Al_2_O_3_)/P_2_O_5_	MgO (%)	MER^a^	Cd (ppm)	*X*_Cd_ (mg Cd/kg P_2_O_5_)
1	0	1	3.55	2.02	5.47	2.11	1.02	2.40	2.900	81.690
2	0.05	0.95	5.42	1.92	5.21	1.31	0.98	1.50	2.756	50.821
3	0.1	0.9	7.29	1.82	4.94	0.93	0.95	1.06	2.611	35.799
4	0.15	0.85	9.17	1.72	4.67	0.70	0.91	0.80	2.467	26.913
5	0.2	0.8	1.04	1.62	4.41	0.55	0.88	0.63	2.323	21.041
6	0.25	0.75	1.91	1.52	4.14	0.44	0.85	0.50	2.179	16.872
7	0.3	0.7	1.78	1.41	3.87	0.36	0.81	0.41	2.034	13.759
8	0.35	0.65	1.66	1.31	3.61	0.30	0.78	0.34	1.890	11.346
9	0.4	0.6	1.53	1.21	3.34	0.25	0.74	0.29	1.746	9.21
10	0.45	0.55	2.40	1.11	3.07	0.21	0.71	0.24	1.601	7.849
11	0.5	0.5	2.27	1.01	2.81	0.17	0.67	0.20	1.457	6.541
12	0.55	0.45	2.15	0.91	2.54	0.14	0.64	0.17	1.313	5.437
13	0.6	0.4	2.02	0.81	2.27	0.12	0.61	0.14	1.168	4.491
14	0.65	0.35	2.89	0.71	2.01	0.10	0.57	0.12	1.024	3.672
15	0.7	0.3	29.76	0.61	1.74	0.08	0.54	0.10	0.880	2.956
16	0.75	0.25	31.64	0.51	1.48	0.06	0.50	0.08	0.736	2.325
17	0.8	0.2	33.51	0.40	1.21	0.05	0.47	0.06	0.591	1.764
18	0.85	0.15	35.38	0.30	0.94	0.04	0.43	0.05	0.447	1.263
19	0.9	0.1	37.25	0.20	0.68	0.02	0.40	0.03	0.303	0.812
20	0.95	0.05	39.12	0.10	0.41	0.01	0.37	0.02	0.158	0.405
21	1	0	41.00	0.00	0.14	0.0034	0.33	0.01	0.014	0.034

^a^Minor element ratio, MER = 100 × (∑(Al_2_O_3_, Fe_2_O_3_, MgO))/P_2_O_5_ (%)

## Results and discussion

Statistical analysis allowed the assessment of the optimum composition of SS and MBM to be used in the co-combustion process in terms of quality parameters of the obtained ashes. Characteristic parameters of ashes after combustion of MBM and SS mixtures are shown in Table 3.

**Fig. 2:**
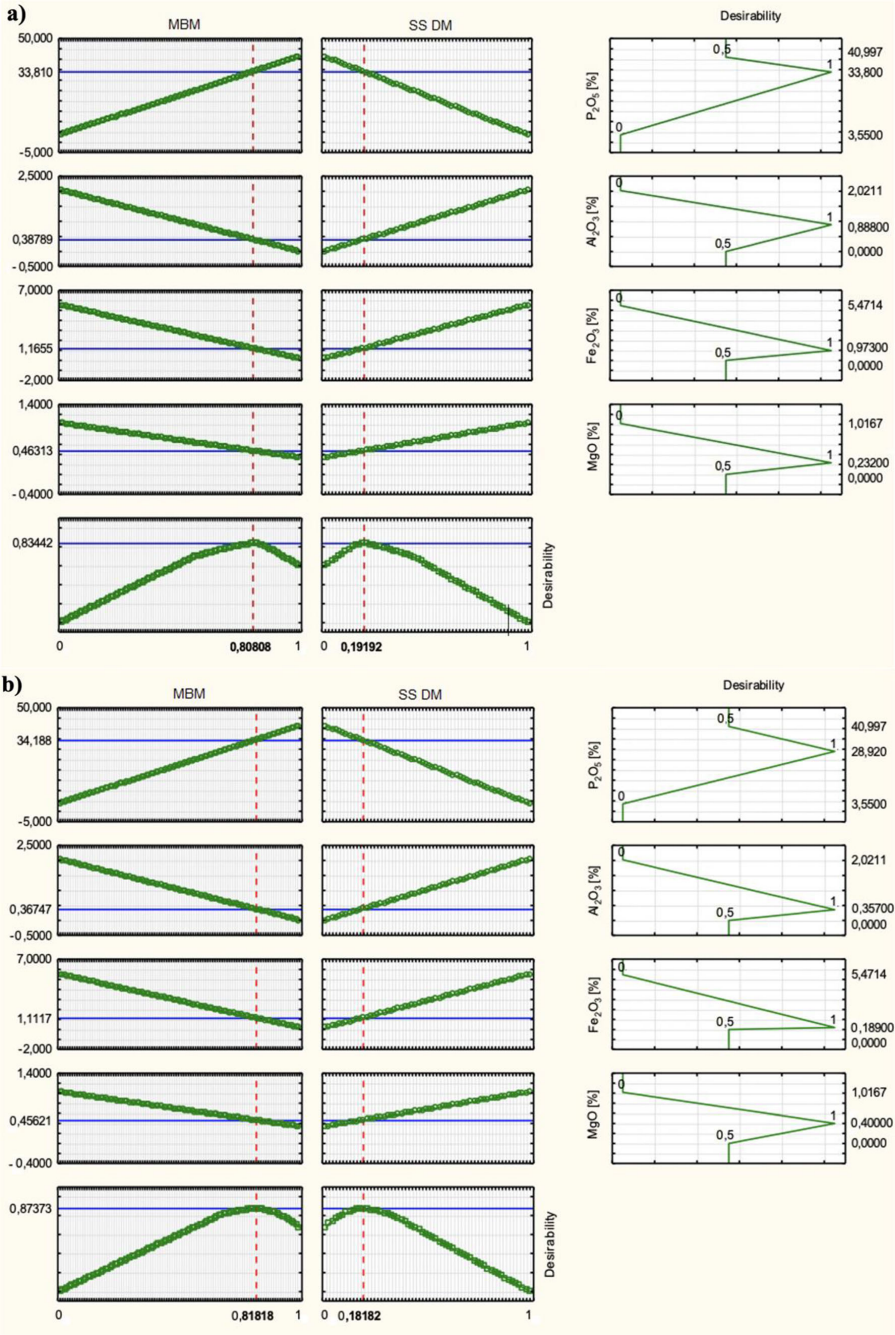

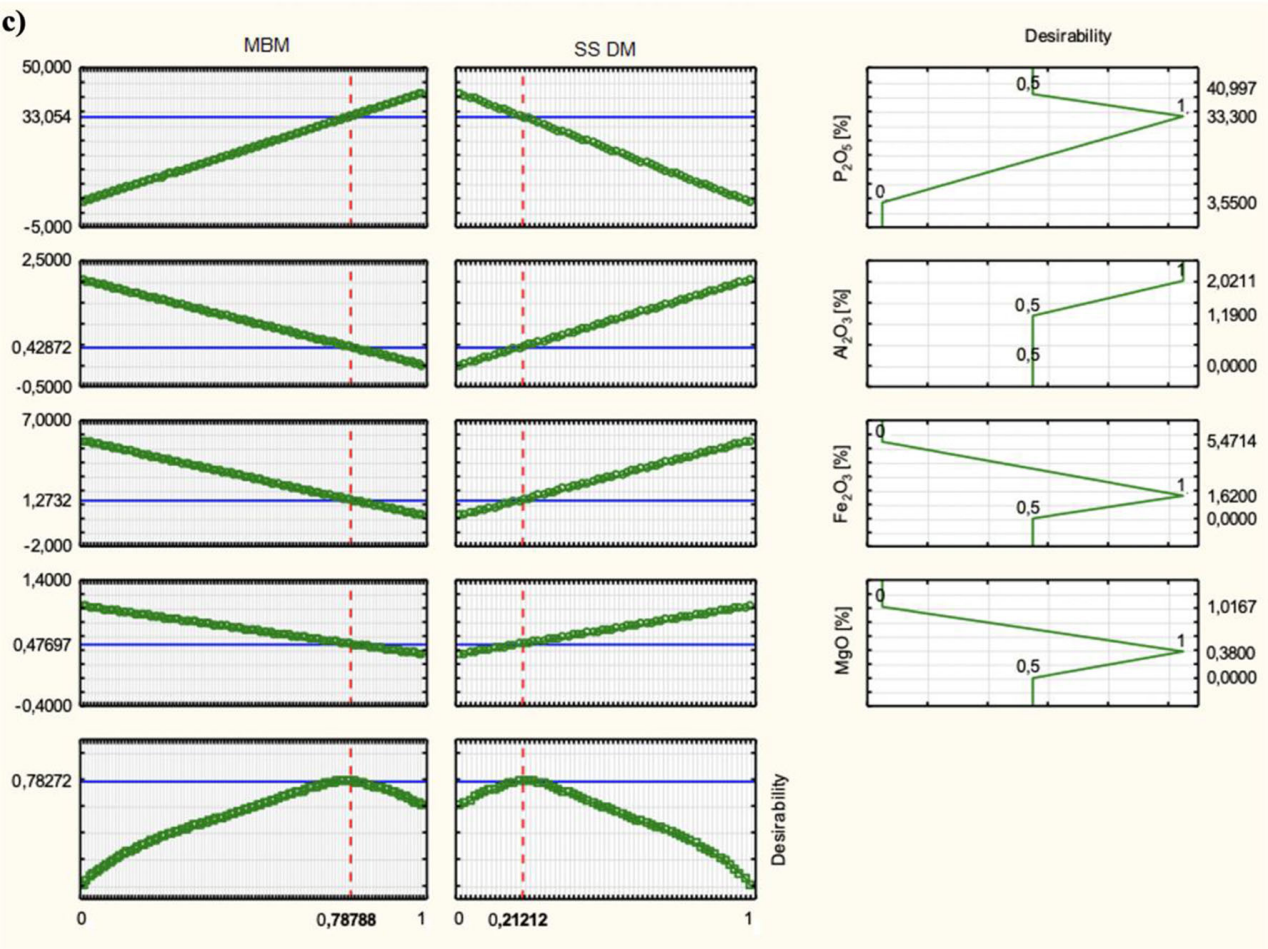
The utility function with the predictive model. **a** Morocco–Togo. **b** Tunisia–Syria. **c** Senegal LamLam

Model coefficients determined according to Eq. 4 were as follows: Y(P_2_O_5_) *b*_1_—40.997; *b*_2_—3.550; Y(Al_2_O_3_) *b*_1_—0; *b*_2_—2.021; Y(Fe_2_O_3_) *b*_1_—0.143; *b*_2_—5.471; Y(MgO) *b*_1_—0.332; *b*_2_—1.017. The results confirm the linear form of the models. To select most favourable variables with regard to the expected values (Table 1), utility function values of the independent variables were used, where a value of 1 means most favourable and 0 unfavourable. Examples of utility functions with a predictive model are shown in Fig. 2.

The analysis of co-combustion of MBM and SS mixtures was made in terms of assessing the quality of ashes obtained to compare their quality and the quality of phosphorites. Expected and approximated parameters of ashes (MBMA and SSA) from the combustion of their mixtures of are shown in Table 4. BPL value of these ashes could be considered to represent a high-quality material, with BPL in the range 71–76 (only one value of 66).

**Table 4:** Expected and approximated parameters of ashes (MBMA and SSA) from the combustion of mixtures of MBM and SS

Phosphorite	Expected parameters (%) as in phosphorite							Approximated parameters in MBMA and SSA mixtures								
	Value (%)						(Fe_2_O_3_ + Al_2_O_3_)/P_2_O_5_	Mass participation		MBMA + SS (%)						(Fe_2_O_3_ + Al_2_O_3_)/ P_2_O_5_
	P_2_O_5_	Al_2_O_3_	Fe_2_O_3_	MgO	BPL	MER^a^		MBM	SSDM	P_2_O_5_	Al_2_O_3_	Fe_2_O_3_	MgO	BPL^b^	MER^a^	
Morocco–Togo	33.8	0.888	0.973	0.232	74	6.2	0.06	1.0	0.238	33.81	0.388	1.166	0.463	74	6.0	0.05
Tunisia–Jordan	29.84	0.423	0.287	0.328	65	3.5	0.02	1.0	0.269	33.05	0.429	1.273	0.477	72	6.6	0.05
Morocco–Syria	29.65	0.348	0.171	0.393	65	3.1	0.02	1.0	0.208	34.57	0.347	1.058	0.449	76	5.4	0.04
Tunisia–Syria	28.92	0.357	0.189	0.4	63	3.3	0.02	1.0	0.222	34.19	0.367	1.112	0.456	75	5.7	0.04
Tunisia–Syria	28.87	0.427	0.197	0.44	63	3.7	0.02	1.0	0.269	33.05	0.429	1.273	0.477	72	6.6	0.05
Syria	30.0	0.23	0.17	0.45	66	2.8	0.01	1.0	0.208	34.57	0.347	1.058	0.449	76	5.4	0.04
Tunisia	29.1	0.47	0.36	0.65	64	5.1	0.03	1.0	0.302	32.30	0.470	1.381	0.491	71	7.3	0.06
Algiers	29.9	0.32	0.44	0.95	65	5.7	0.03	1.0	0.253	33.43	0.408	1.219	0.470	73	6.3	0.05
Togo	33.5	1.08	1.33	0.17	73	7.7	0.07	1.0	0.285	32.68	0.449	1.327	0.484	71	6.9	0.05
Morocco	31.6	0.3	0.2	0.45	69	3.0	0.02	1.0	0.208	34.57	0.347	1.058	0.449	76	5.4	0.04
Egypt	28.9	0.49	1.7	0.3	63	8.6	0.08	1.0	0.414	30.03	0.592	1.704	0.532	66	9.4	0.08
Senegal Matam	32.3	1.19	0.8	0.38	71	7.3	0.06	1.0	0.302	32.30	0.470	1.381	0.491	71	7.3	0.06
Senegal LamLam	33.3	3.59	1.62	0.38	73	16.8	0.16	1.0	0.269	33.05	0.429	1.273	0.477	72	6.6	0.05

^a^Minor element ratio, MER = 100 × (∑(Al_2_O_3_, Fe_2_O_3_, MgO))/P_2_O_5_ (%)

^b^BPL, bone phosphate of lime = 2.1852 ^.^ P_2_O_5_

Levels of impurities (Fe_2_O_3_ + Al_2_O_3_)/P_2_O_5_ in phosphorites were in the range 0.01–0.08 (only one value of 0.16) and in MBMA and SSA mixtures in the range 0.04–0.08. The acceptable level of impurities in H_3_PO_4_ used to produce fertilisers is 0.02–0.08 (Podolak 2017). MER values for phosphorites were in the range 3.0–16.8 and for MBMA and SSA mixtures in the range 5.4–9.4.

### Environmental and economic effect of the co-combustion of MBM and SS mixtures

An actually developed MBM combustion project realised by the Farmutil Company (Kowalski and Makara 2017) assumes a combustion of 30,000 t/year of MBM and the production of 7500 t/year of ash containing hydroxyapatite. As mentioned, 250 kg of ash was obtained from 1 t of combusted MBM and 280 kg of ash from 1 t of combusted SSDM.

The quality parameters of ash from the co-combustion of MBM and SS were calculated for two variants assuming the co-combustion of a mixture of 1000 kg MBM and 269 kg SSDM (variant I) or 1000 kg MBM and 83 kg SSDM (variant II). Variant I value resulted from the last line of Table 4. This is proportion allowing to obtain ash containing 21.2% SSDM, comparable with phosphorite Senegal LamLam. Variant II value resulted from a total of 2500 t/year SSDM of SS produced by the company (Kowalski and Makara 2017). The co-combustion of a mixture of 30,000 t/year MBM and 2499 t/year SS could allow the utilisation of all the SS produced by the company per year.

In variant I for the capacity of combustion mixture of 30,000 t/year MBM and 8070 t/year SSDM could be produced 9870 t/year of hydroxyapatite ash qualitatively comparable with phosphorite Senegal LamLam characterised with BPL—72; (Fe_2_O_3_ + Al_2_O_3_)/P_2_O_5_—0.05; and MER—6.60.

In proposed for implementation in Farmutil variant II, for the capacity of combustion mixture of 30,000 t/year MBM and 2.499 t/year SSDM could be produced 8200 t/year of very high-quality ash characterised with BPL—83; (Fe_2_O_3_ + Al_2_O_3_)/P_2_O_5_—0.04; and MER—2.85.

The environmental effect is the substitution of 8200 t of phosphorites used for the production of phosphoric fertilisers with ashes from the co-combustion of a mixture of MBM and SS. Ultimately, this amount may increase to over 27,000 t if the amount of thermal MBM utilisation increases to 100,000 t/year. The proposed method also allows the utilisation of all the SS obtained in Farmutil, an amount of 2500 t/year SSDM after biological treatment of wastewater from MBM and municipal production.

The high-quality ashes (Kowalski and Makara 2017) do not contain fluorine compounds, and during their processing into phosphoric acid, there is no need for a complicated phosphorite de-fluorination stage. Cadmium content is also at trace levels. The ashes also do not contain radioactive compounds, which are present in trace amounts in all phosphates. For example, Senegal phosphorite contains 101 ppm U_3_O_8_ (BAT 2000).

The co-combustion of MBM and SS is an example of a production complex using cleaner technologies and the incorporation of a number of elements included in the principles of a circular economy CE on a microeconomic scale. The analysed CE concept is based on the use of cleaner production CP activities such as the recycling (in- and off-process) of substrates used for combustion, the substitution of natural phosphorites in the production of phosphorus compounds by ash and its use for technological purposes (Stokłosa et al. 2019; Kowalski and Krupa-Żuczek 2007).

The economic effects of selling ash from MBM and SS mixtures are also significant. A developed capacity of 8200 t/year of ashes amounts to 1.033 million USD and a target capacity of 27,330 t/year ashes to 3.443 million USD. The price adopted for the calculation corresponds to the current price of phosphorites on the Polish market of 126 USD/t phosphorites, loco Polish harbour (based on Money.pl). There are at least four potential recipients of this product on the Polish market.

## Conclusions

Based on the results, the following conclusions can be drawn:

1. The optimal mass proportions of co-combusted MBM and SS to obtain ash with a quality comparable or better than that of phosphorites used for the production of phosphoric acid were determined. Maximum SSDM/MBM ratio to 0.269 allowed obtaining ashes, considered to be a high-quality phosphate raw material with average BPL value of 72.0, level of impurities (Fe_2_O_3_+ Al_2_O_3_)/P_2_O_5_ of 0.05 and MER of 6.6.
2. In the case of the developed project analysed, the goal was the co-combustion of a mixture of 30,000 t/year MBM and 2499 t/year SS, which would allow the use of all the SS produced by Farmutil Co. The very high-quality ash obtained had a BPL value 83, a level of impurities (Fe_2_O_3_+ Al_2_O_3_)/P_2_O_5_ of 0.04 and an MER value of 2.85.

## Data Availability

All data generated or analysed during this study are included in this published article.
